# Surgical Treatment of Perianal Fistulizing Crohn's Disease: From Lay-Open to Cell-Based Therapy—An Overview

**DOI:** 10.1155/2014/146281

**Published:** 2014-11-06

**Authors:** Gianluca Pellino, Francesco Selvaggi

**Affiliations:** Unit of General Surgery, Second University of Naples, Piazza Miraglia 2, 80138 Naples, Italy

## Abstract

*Background*. Perianal Crohn's disease (CD) can be challenging. Despite the high incidence of fistulizing CD, literature lacks clear guidelines. Several medical, surgical, and combined treatment modalities have been proposed, but evidences are scarce. *Methods*. We searched the literature to assess the facets of perianal CD, with particular focus on complex fistulae. Disease epidemiology, classification, diagnosis, activity scoring systems, and medical-surgical treatments were assessed. *Results*. Perianal fistulizing CD is common, frequently associated with upper gastrointestinal and colorectal CD. Complex fistulas often require repeated treatments. Continence is a major concern when dealing with repeated procedures. A prudent pathway is to resolve active sepsis and to limit damages, delaying a definitive treatment to the time when acute phase has been controlled. The improved diagnostic techniques allow better preoperative planning and are useful in monitoring the response to treatment. Besides newer devices, cell-based treatments are promising tools which have recently enriched the treatment portfolio. However, the need for proctectomy is still disturbingly high in CD patients with complex perianal fistulae. *Conclusions*. Perianal CD can impair quality of life and lead to need for proctectomy. A staged approach is reasonable. Treatment success can be improved by multimodal treatment and collaborative management by experienced gastroenterologists and surgeons.

## 1. Introduction

Approximately 40–60% of Crohn's disease (CD) patients have perianal involvement, and 30% have perianal fistula [1, 2]. The pathogenesis of perianal fistulae in CD is different from that of cryptoglandular ones. Usually, fistulae are believed to originate from either deep penetrating ulcers or anal gland infection/abscess [3]; however, other theories have been advocated in CD patients, involving microbiological, immunological, and genetic factors [4, 5]. These observations are in agreement with the higher rate of high, complex fistulae observed in CD patients with active rectal disease [6]. It is well known that CD is an independent risk factor for postoperative septic complications [7], suggesting the relevant contribution of intestinal microbiota to such a mechanism. Genetic and epigenetic factors play a pivotal role in CD, as the risk of developing CD and its related complications is higher in relatives of CD patients than in general population [5, 8]. Genetic susceptibility is suggested by the frequent association between perianal CD and colorectal as well as upper gastrointestinal disease involvement [9, 10] so that CD is a different entity from penetrating abdominal CD [9] and is also associated with worse prognosis in the long term [11]. Also, as active, persisting disease leads to fibrosis in CD [5], a hypoxia-mediated mechanism could also play a role [12]. Epidemiology of perianal CD should be considered in the light of the presumed incidence and prevalence of the disease per country. In Italy, the estimated incidence and prevalence of CD are reported to be as high as 5/100 000 inhabitants/year and 59.63/100 000 inhabitants, respectively [13]. In other words, a prevalence of nearly 11 000 patients with CD perianal fistulae can be predicted in Italy. Also, it has been reported that in 10% of patients perianal fistulae can be the first manifestation of CD [14]. This means that approximately 300 patients per year will present with perianal fistulae before receiving diagnosis of CD in Italy. These data give an insight of the burden of disease and highlight the importance of knowing how to manage such patients in the acute settings to avoid subsequent problems. Repeated maneuvers and too aggressive approaches justify the rate of incontinence still observed with conventional, cutting techniques in these patients, even reaching 50% for intersphincteric fistulae [15]. It should be noted that faecal diversion and proctectomy still play a relevant role in perianal CD [10, 16–19], further suggesting that the ideal management of such patients is yet to be achieved.

We present the most recent advances of surgical treatment of perianal CD in the light of the new discoveries in medical treatment, moving from lay-open to cell-based therapy, as well as imaging techniques.

## 2. Classification and Preoperative Assessment

The Montreal revision of Vienna classification identified perianal disease as an additional category (identified as “*p*” added to CD behavior) to be considered in association with the three main patterns of disease (penetrating; inflammatory; nonpenetrating noninflammatory) [20, 21]: this confirms that perianal CD is distinct entity from abdominal fistulizing CD [9, 11] and can occur in association with and independently of CD baseline behavior.

Sir Parks in 1976 classified perianal fistulae as intersphincteric, transsphincteric, suprasphincteric, extrasphincteric, and superficial, according to their relations with the external sphincter [22]. Several classifications were subsequently proposed for perianal CD, among which the Cardiff classification is one of the most known. However, it is considered difficult to apply in routine practice and of limited interest in terms of patient management [23]. The technical review published in 2003 by the American Gastroenterological Association (AGA) proposed a simpler classification, widely adopted, identifying fistulae as either “simple” (low, with a single external opening) or “complex” (high, may have ≥1 external opening, associated with perianal abscess, rectovaginal fistula, anorectal stenosis, or active rectal disease) [1]. However, when planning treatment, each patient needs to be evaluated in detail, in order to avoid inappropriate treatment or overtreatment (i.e., a low, anovulvar fistula amenable with fistulotomy is classified as “complex”), suggesting that also such classification has grey areas.

Concerning the assessment of perianal disease, the most widespread tool is the perianal disease activity index (PDAI) [24], a clinical score assigning 0 (none) to 5 (highest) points to each of the following: fistula discharge, pain, restriction of daily activity, restriction of sexual activity, type of perianal disease, and degree of induration.

Physical examination must be implemented with endoscopy and at least one among examination under anesthesia (EUA), endoanal ultrasonography (EUS), and magnetic resonance imaging (MRI) [1, 25, 26]. The effectiveness of EUS in pediatric patients has recently been reported, and this is relevant when considering the high rate of patients being observed with inflammatory bowel disease in the developmental age [27–29]. Concerning EUS, this technique has now been implemented with 3D image reconstruction, allowing better identification of the relationship between the fistula and the sphincter complex. In a series of 85 patients, Reginelli et al. showed that 3D-EUS provides accurate anatomical information and suggested that minor defects can be recognized, not detectable with conventional EUS [30]. In addition, the technique can be made more accurate by instillation of hydrogen peroxide. West et al. [31] compared the efficacy of hydrogen peroxide enhanced 3D-EUS with endoanal MRI in a prospective cohort of 21 patients with perianal fistula and reported an agreement as high as 80% in identifying the primary track for 3D-EUS and surgery, 90% for both MRI and surgery, and 3D-EUS and MRI. 3D-EUS was as accurate as MRI, in identifying the internal opening (86% both 3D-EUS and surgery, and MRI and surgery, 90% 3D-EUS and MRI) [31]. These findings support the reliability of both endoanal 3D-EUS and MRI in preoperative evaluation of fistula-in-ano. However, care must be paid not to overlook distant abscess, better visualized with conventional MRI. Furthermore, it is important to avoid errors with 3D-EUS originating from stitches or setons, which can simulate abscesses after enhancement with hydrogen peroxide [32].

Imaging techniques have also been reported to be useful in guiding the patient management and to assess response to treatment [27, 33, 34]. A meta-analysis [35] comparing MRI and EUS for the evaluation of perianal fistulae showed a slight superiority of the former; however comparable results can be expected in experienced hands, and performing a combination of two modalities among EUA, EUS, and MRI may reach 100% accuracy.

## 3. Treatment

### 3.1. Acute Presentation: Control of Sepsis

Up to 60% of patients with perianal CD shall present with an abscess requiring drainage [36, 37]. Literature lacks good quality studies on antibiotic treatment alone for perianal abscess and fistulae in CD, but most agree that a clinical response is observed after 6 to 8 weeks and mainly consists of reduced discharge, while fistula closure is uncommon and symptom recurrences are highly probable [38, 39]. As now, antibiotics (ciprofloxacin or metronidazole [39, 40]) can be considered first-line treatment, but surgery should be considered if symptoms worsen or are unacceptable and if a response is not observed within 6–8 weeks. Thia et al. [40] randomized 25 patients with perianal CD into three treatment arms: ciprofloxacin (10), metronidazole (7), and placebo (8). At 10-week follow-up, remission occurred in 30%, 0, and 12.5% of patients receiving ciprofloxacin, metronidazole, and placebo (*P* = 0.41). Response occurred more frequently in patients treated with ciprofloxacin but the differences were not significant. Once indication to surgery is made, aims of treatment of the acute phase are to drain adequately the abscess and to avoid sphincter lesions. Michelassi et al. [36] showed that incision and drainage achieved healing in 2/3 of 34 patients with perianal CD, while 1/3 subsequently presented with fistula. Others have reported that almost half of patients with perianal CD abscess will subsequently need treatment for associated fistulae [41]. Pritchard et al. [41] treated 38 consecutive CD patients presenting with perirectal abscess, of whom 30 had simple and eight horseshoe abscesses. Fifty-three percent of patients underwent incision and drainage, whereas 47% had drain catheters placed. After abscess resolution, abscesses recurred in 45% and 56% of the patients who underwent catheter drainage and incision and drainage, respectively [41]. It is common practice in some surgical teams to place a mushroom (or Malecot) catheter to drain large cavities, but it is mostly done following empirical principles [14]. Should a low, intersphincteric fistula be found at surgery, spontaneous healing is observed in approximately 35% of patients, while fistulotomy achieves complete healing in 60–100% of patients [36, 42, 43]; it is prudent and recommended to place loose-setons along fistulae for which the relations with anal sphincters are unclear or in those extending upward. The surgeons should carefully check that external opening is wide enough to ensure adequate draining; primary suturing of potential residual cavities is proscribed.

Once sepsis is controlled, fistula assessment is recommended by means of MRI or EUS, should it have not been performed before surgery.

### 3.2. Maintenance/Preparation

Once sepsis is controlled, it is important to maintain the remission, keeping the site drained. The commonest strategy is represented by atraumatic, loose-seton placement (silastic or ethibond), aimed at preventing abscess formation and to avoid sphincter section. This is a safe procedure to limit damages, and short-time healing is achieved in 48–100% of patients [44]. No accepted data are available concerning the ideal time to remove the seton, and this is performed on empirical basis, reported to range between 3 and 58 months by some authors [33]. If an early removal may intuitively lead to abscess formation, a prolonged stay in situ can result in fibrosis of the fistulous track, leading to persistent incapability to heal after seton removal. Furthermore, disappointing results can be expected in the long term, with symptomatic recurrences occurring in over 80% of patients after removal [33]. However, placing a seton loosely is a safe and useful strategy before attempting a definitive approach, without continence disturbances.

In the eventuality of active disease not amenable with conservative treatment, a fecal diversion may be needed and usually restores patient well-being rapidly [45]. In a study of 79 patients with severe, debilitating CD undergoing faecal diversion with loop-ileostomy, 91% had clinical improvement and allowed delaying definitive surgery at a later stage, under more appropriate circumstances [45]. On the other hand, one should consider that diverted CD patients are unlikely to undergo stoma reversal, with more than 80% of patients receiving an indefinite diversion [17]. This also raises safety concerns, due to the presence of active disease with consequent higher risk of malignancies [46]. Aiming to identify predictors of definitive stoma, Galandiuk et al. [47] reviewed the clinical data of 356 consecutive patients with CD, of whom 86 were with perianal CD. Active colonic disease, anorectal stenosis, and multiple perianal procedures were associated with the need of permanent diversion [47].

### 3.3. Definitive Treatment

Low/simple fistulae are well treated with tissue separating techniques, as fistulotomy achieves almost 100% of healing with minimal risk of continence disturbances [36, 48, 49]. Tissue separating techniques can be carried out at the time of seton removal in selected patients for complex fistulas, but the risk of incontinence is a major issue in such an eventuality [6, 50].

More conservative treatments have consequently been proposed. The efficacy of infliximab (IFX, a murine/human chimeric monoclonal antibody directed toward TNF-*α*) in inducing complete healing in CD perianal fistulae has been reported to be as high as 46% after induction therapy (3 infusions at weeks 0, 2, and 6) [51], as well as the utility of establishing a maintenance regimen. In fact, in the ACCENT II trial, 36% of patients receiving scheduled maintenance IFX effusions had complete healing confirmed after 54 weeks, compared with 19% in the placebo group [52]. In order to increase the rate of success Topstad et al. [53] proposed a combined approach, consisting of surgery aimed at draining sepsis with seton placement, followed by IFX effusions. The good results obtained were confirmed by others [54, 55], showing higher rate of response and lower recurrences with EUA plus IFX than with IFX alone [54], and a decrease of PDAI after EUA plus IFX [55]. A study comparing three groups treated with IFX, surgery, or combined treatment showed that the former had shorter time to heal and longer time to recur [56]. However, IFX administration can have significant side effects and is contraindicated in patients with abdominal fibrostenosing CD [1, 57]. Aiming to reduce systemic effects and to treat patients with contraindications to intravenous administration, Poggioli et al. proposed injection of IFX at the fistula site [58] and showed complete healing in 10 out of 15 patients treated (67%). The same promising results were confirmed using another biological drug, adalimumab (ADA), a fully humanized anti-TNF-*α* antibody [59]. The drawback of this approach is the local fibrosis caused by the drugs, but it seemed less marked with ADA [59].

Advancement flaps of rectal mucosa represent another surgical option for the management of complex perianal and rectovaginal fistulae (RVF). The advantages of flap procedures consist of both avoidance of external wounds, the healing of which could be impaired by active sepsis and contribute to perineal scarring, and reduced manipulation of the sphincters, with lower risks of incontinence. Flaps are contraindicated with active proctitis. The procedure is easier in patients with perineal descent and internal intussusception. However, midterm success rates do not exceed 57% [60, 61]. CD is an independent predictor of failure [60, 61], with a hazard ratio of 2.92 versus patients with cryptoglandular fistulae [60]. RVF can be approached for flap procedures either transanally or tranvaginally. A systematic review of 11 studies reporting on 224 flap procedures for RVF in CD patients showed that pooled primary closure (53% versus 61% transrectal versus transvaginal) and pooled overall closure (75% versus 81%, transrectal versus transvaginal) were similar with both approaches [62]. Very recently a new technique was proposed to enhance the outcomes of flap repair for complex CD fistulae, combining it with video-assisted anal fistula treatment (VAAFT) [63]. Out of 11 patients in whom the treatment was completed, 9 had a complete response at 9-month follow-up (82%), with no continence disturbances.

Gingold et al. [64] performed the ligation of the intersphincteric fistula track (LIFT) procedure in 15 consecutive patients with complex fistulae, reporting healing of the LIFT site in 8 of 12 patients (67%) with 12-month follow-up. The authors suggested that lateral versus midline location (*P* = 0.02) and longer mean fistula length (*P* = 0.02) were predictors of 12-month LIFT site healing [64]. No patients experienced incontinence.

**Figure 1:**
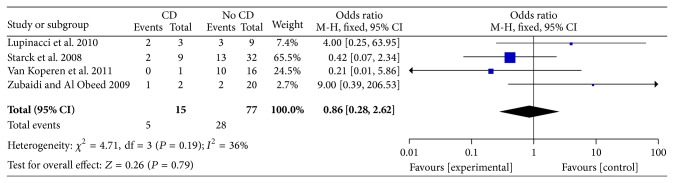
Forest plot of the failure (event) of patients undergoing plug procedure with (CD) or without (no CD) diagnosis of Crohn's disease. Only papers in which CD could clearly be identified and only patients with complex fistulae were included. No differences were observed between Crohn's disease patients and controls (OR 0.86, 95%CI 0.28–2.62, *P* = 0.79) (Mantel-Haenszel fixed effect). Low heterogeneity is observed: *I* ^2^ = 36%.

Less invasive strategies have been also attempted with fistula track fillers, namely, plugs and glues. A systematic review of 20 studies aimed at comparing the results of bioprosthetic anal fistula plug in patients with CD compared with non-CD patients has recently been published. The authors suggested that studies were too heterogeneous to attempt meta-analysis [65] but reported an overall pooled fistula closure of 55% (22/42 patients) and 54% (265/488 patients) in CD and non-CD, respectively. As the authors included both complex and simple fistulae, we tried to assess the failure rate by only evaluating data of patients with complex fistulae and from studies where the diagnoses were clearly reported. By including 4 studies [66–69], we found a rate of no response in 33% versus 36% in CD versus non-CD patients, but this slight difference was not statistically relevant and 36% heterogeneity (assessed with *I* ^2^) was observed (Figure 1).

Concerning treatment with glues, literature lacks good quality studies focused on CD patients. An open label, randomized, controlled trial from the Groupe d'Etude Thérapeutique des Affections Inflammatoires du Tube Digestif (GETAID) on CD patients comparing fibrin glue with observation only showed clinical remission in (13/34) 38% of glue group compared with 6/37 (16%) in controls at 8-week follow-up [70]. Twenty controls were also treated with fibrin glue, and 9 maintained remission after 16 weeks. Considering all 54 patients receiving fibrin glue, only 11 (20%) were in remission at last follow-up (median 37 months for fibrin glue ab initio and 17 months for the crossover group) [70]. In published series, treatment success ranges between 0 and 100% [70–73].

Caution must be paid when trying to interpret data on glues and plugs [65, 74]: many authors have conflict of interests; devices are very expensive; patients, procedures, and studies are heterogeneous; the follow-up is often too short; healing is assessed only clinically; no postoperative MRI scan is performed in any study, although some assess the patients with MRI preoperatively [70]. However, limited sphincter manipulation is needed, with theoretically no risk of incontinence so that the procedures can be harmlessly repeated.

Aiming to improve the rate of success of fibrin glue, modified glue formulations have been proposed. Garcia-Olmo et al. [75] randomized 49 patients (14 suffering from CD) with perianal fistulae to treatment with either adipose derived stem cells (ASCs) in fibrin glue or fibrin glue alone and reported an increase in healing rate from 18% with fibrin glue alone to 71% in patients receiving the glue added with ASCs [75]. This publication paves the way to the so-called cell-based treatment of perianal fistulae. ASCs are living adult stem cells of mesenchymal origin which are activated in an inflamed environment (e.g., fistulae). ASCs can simultaneously regulate multiple upstream pathways of inflammation. These cells are activated by IFN-*γ* released in inflamed areas and have the capability to suppress both the proliferation of activated lymphocytes and the production of inflammatory signals through the expression of indoleamine 2,3-dioxygenase [76, 77]. These effects ultimately lead to elimination of activated lymphocytes and proinflammatory cytokines, resulting in pain cessation and tissue repair. This perspective, if confirmed, is fascinating when dealing with CD patients with complex perianal fistulae, as the principle of ASCs relies on stimulating the host immune system to almost physiologically remove the source of inflammation, with no reported side effects and limited perineal scarring. A phase-III multicentric, randomized, controlled trial is currently recruiting CD patients with complex perianal fistulae unresponsive to conventional medical or surgical treatment, investigating the efficacy of allogenic ASCs as intralesional injection versus placebo, saline solution (ADMIRE-CD, registered as NCT01541579 at clinicaltrial.gov). This is a double-blind trial, and healing is going to be assessed both clinically and by means of MRI (with central blind assessment) up to 52 weeks after treatment. Final data collection date should be around January 2015. If the results of this study will confirm the enthusiastic findings of prior studies with ASCs as filling devices, it will add a useful tool to the armamentarium of surgeons and physicians dealing with complex perianal CD.

In refractory perianal disease with concomitant active proctitis unresponsive to medical/surgical treatment, a faecal diversion can be necessary. Since less than 20% of diverted CD patients will undergo stoma reversal, this should be considered a last resort [17, 18]. It has been also reported that 20% of patients receiving colectomy will require proctectomy within 5 years [19]. These observations justify the disturbingly high rate of proctectomy still observed, ranging between 10 and 18% [10, 49]. Recently, IFX proved to be effective in CD patients with failed ileorectal anastomosis candidates to proctectomy, preserving the rectum in 10/12 patients (83.3%) [78]. Proctectomy is still to be favored over indefinite diversion.

## 4. Conclusions

**Figure 2:**
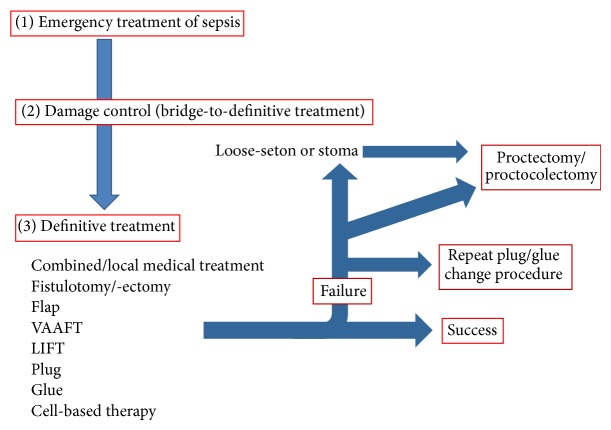
A proposed algorithm to manage patients presenting with perianal Crohn's disease. In patient needing immediate drainage of abscess, emergency treatment is performed, aimed at controlling sepsis (1). Should associated fistulous tracks be identified, it is prudent to place loose-seton(s) as bridge-to-definitive treatments, aiming to maintain the drainage, avoiding abscess formation. Patients with very active disease may require temporary faecal diversion (2). Once sepsis is controlled and the patient is in good general health status, definitive treatment can be attempted, consisting of either tissue separating techniques (fistulotomy, fistulectomy) or more conservative and combined approach (3). An interval of 2-3 months seems acceptable. In patients with failure, procedures can be repeated, favoring approaches which do not increase significantly the risk of incontinence. Stoma or proctectomy may be required in refractory, frail patients. LIFT: ligation of the intersphincteric fistula track, VAAFT: video-assisted anal fistula treatment.

Despite perianal fistulae affecting a relevant rate of patients suffering from CD, literature lacks evidence-based pathways for the management of complex perianal fistulae in CD. This is commonly performed with empirical approaches. Also, unlike ulcerative colitis [29, 79] and colorectal surgery [80], little is known concerning treatment according to age, as this may be relevant when balancing advantages and potential side effects of medical compared with surgical treatment. Furthermore, the risk of cancerogenesis and a potential role of timely surgery in removing inflammation ultimately reducing the risk of cancer are less investigated in CD than in ulcerative colitis patients [46, 81]. When dealing with perianal CD, it is pivotal to assess the entire patient condition and to careful balance medical and surgical treatment. A staged approach, as reported in Figure 2, may be a prudent choice.

## Abbreviations

ADA: Adalimumab
AGA: American Gastroenterological Association
ASCs: Adipose derived stem cells
CD: Crohn’s disease
EUA: Examination under anesthesia
EUS: Endoanal ultrasonography
GETAID: Groupe d’Etude Thérapeutique des Affections Inflammatoires du Tube Digestif
IFX: Infliximab
LIFT: Ligation of the intersphincteric fistula track
MRI: Magnetic resonance imaging
PDAI: Perianal disease activity index
RVF: Rectovaginal fistula
VAAFT: Video-assisted anal fistula treatment.
